# Biliary Nephritis

**Published:** 1908-10-10

**Authors:** 


### BILIARY NEPHRITIS.

The infective complications that may result at a distance from the liver which is the site of bacterial cholangitis are numerous enough. Endocarditis is fairly common; meningitis is possible; there is scarcely an organ that may not be attacked. Nevertheless, very little stress has hitherto been laid upon the effects of cholangitis upon the kidneys. Slight albuminuria is sometimes mentioned, but the possibility of a true nephritis of cholangitic origin is often overlooked. Mollière, it is true, has described cases of cirrhosis with lithiasis in which, from septic absorption, acute nephritis resulted, the post-mortem examination revealing typical large white kidneys. It seems worth while to insist more strongly upon the possibility of this renal lesion ensuing upon infective cholangitis, whether the latter is accompanied by jaundice or not. In a case already described in a former issue the nephritis developed quite late in the course of the hepatic disease, but it became so severe that the urine contained upwards of 13 parts per 1,000 of albumen, equivalent in the case in question to nearly an ounce of albumen every twenty-four hours.

The patient was ill in bed at the time the albuminuria developed, and no generalised œdema resulted. This recalls what happens in cases of scarlatinal nephritis; when the latter develops while the patient is ill in bed, œdema is often conspicuous by its absence; it is chiefly in convalescing cases, when the patients are up and about, that œdema attracts attention to the state of the kidneys.

The question arises as to how the nephritis comes about. Is it due to microbial elimination, or is it due to the action of a toxin? The bacillus coli communis has been recovered in pure culture both from the bile in the gall-bladder and from the urine on more than one occasion. One is familiar with the fact that various microbial diseases may give rise to acute non-suppurative nephritis; the latter may be associated with pneumococci in cases of lobar pneumonia or empyema; and streptococci have been recovered from the inflamed kidneys in many fatal cases of scarlet fever. Possibly, therefore, this biliary nephritis is nothing more than a metastatic infection of the kidneys by organisms. It is, however, also possible that the organisms in the urine were merely those that were being excreted by the kidneys, and that the latter were really affected less by the organisms themselves than by the toxins carried from the liver in the blood-stream. It is quite clear, anyway, that when the gall-bladder was opened and drained the albuminuria rapidly diminished and disappeared, so that the kidney trouble was directly attributable to the cholangitis. The relief to the nephritis by opening the gall-bladder here is exactly comparable to the relief to scarlatinal Bright's disease experienced after suppurating cervical glands have been opened, or other bacterial foci have been drained. The occurrence of nephritis in such cases as these is, therefore, no contra-indication to operative measures, but quite the reverse.

One more case may emphasise this. A female patient, aged thirty-eight, had suffered from jaundice as a child, but had been quite well since that time until the beginning of 1899. She then noticed one day that her eyelids were swollen when she got up in the morning. There was no headache, no vomiting, no ocular trouble, and at the end of the week the œdema had gone. The urine was not examined at this time. Shortly after this, there was a violent attack of biliary colic without jaundice, followed by two other similar attacks later in 1899. On April 1, 1900, there was a fourth bout of biliary colic, very severe, lasting twenty-four hours, recurring on April 4, and leaving behind it a distinct tinge of jaundice. The patient now came into hospital. The liver was felt to be distinctly larger than normal, the spleen was palpable, and the urine contained not only bile pigments but also albumen to the extent of 1 part per 1,000. An operation was performed, and a calculous cholecystitis of quite old date was found; a biliary fistula was left to drain; the liver became its natural size again, and the albuminuria decreased forthwith.

The albuminuria seemed clearly due to a nephritis caused by absorption either of bacilli or of toxins from the infective cholecystitis. There was no other apparent cause for this nephritis. The simplest explanation of the case, therefore, was that the hepatic trouble came first and led to a biliary nephritis, which was relieved when the gall-bladder was opened and drained.

Amongst the complications of infective ascending cholangitis, therefore, must be counted biliary nephritis, susceptible to amelioration or even cure by treatment directed towards the causal infection in the liver.

